# Do health-care institutions perform better under leaders with medical or non-medical backgrounds? A scoping review

**DOI:** 10.1108/LHS-11-2023-0084

**Published:** 2024-05-29

**Authors:** Shazwani Mohmad, Kun Yun Lee, Pangie Bakit

**Affiliations:** Centre for Leadership and Professional Development, Institute for Health Management, National Institutes of Health (NIH), Shah Alam, Malaysia and Seremban District Health Office, Ministry of Health Malaysia, Putrajaya, Malaysia; Centre for Leadership and Professional Development, Institute for Health Management, National Institutes of Health (NIH), Shah Alam, Malaysia

**Keywords:** Leadership, Medical, Health-care institutions, Hospitals, Performance

## Abstract

**Purpose:**

This study aims to summarize studies that compared the performance of health-care institutions led by leaders with medical background versus those with no medical background.

**Design/methodology/approach:**

A systematic search was conducted on three databases: PubMed, Ovid Medline and Google Scholar to identify relevant peer-reviewed studies using the keywords “performance,” “impact,” “physician,” “medical,” “doctor,” “leader,” “healthcare institutions” and “hospital.” Only quantitative studies that compared the performance of health-care institutions led by leaders with medical background versus non-medical background were included. Articles were screened and assessed for eligibility before the relevant data were extracted to summarize, appraise and make a narrative account of the findings.

**Findings:**

A total of eight studies were included, four were based in the USA, two in the UK and one from Germany and one from the Arab World. Half of the studies (*n* = 4) reported overall better health-care institutional performance in terms of hospital quality ranking such as clinical effectiveness and patient safety under leaders with medical background, whereas one study showed poorer performance. The remaining studies reported mixed results among the different performance indicators, especially financial performance.

**Practical implications:**

While medical background leaders may have an edge in clinical competence to manage health-care institutions, it will be beneficial to equip them with essential management skills to optimize leadership competence and enhance organizational performance.

**Originality/value:**

The exclusive inclusion of quantitative empirical studies that compared health-care institutional performance medical and non-medical leaders provides a clearer link between the relationship between health-care institutional performance and the leaders’ background.

## Introduction

The balance of quality versus cost, as well as technology versus humanity, has become extremely complex in the health-care sector, placing increasing demands on doctors’ roles. These issues require excellent leadership to take the helm of health-care institutions (HIs). Doctors were previously regarded as less suitable for leadership roles because of the concern that their background training could have resulted in them becoming “heroic lone healers” (Lee, 2017). The growth of management and the application of new business approaches known as New Public Management in the 1980s pushed this perception even further (Martinussen and Davidsen, 2021). However, the emphasis on patient-centered care and efficiency in clinical outcomes implies that doctors are now increasingly being groomed for leadership roles. Since the global pandemic of COVID-19, there has been a greater emphasis on the significance of effective leadership and management in the health-care sector. HIs all over the world are facing a challenging future due to rising operating costs and higher expectations of the quality of health-care services. To deliver a high-quality health-care service, leader of HI must plan and manage their resources wisely and strategically, besides being the central figures that guide the staff in the everyday operation of the institutions.

In some countries, the majority of medical background leaders work as “hybrid leaders,” managing a clinical workload alongside their management responsibilities (Hamilton *et al.*, 2008; Kippist and Fitzgerald, 2009). This is because the medical field has traditionally been less accepting of doctors who give up clinical work (Opdahl Mo, 2008). In the UK National Health Service (NHS), increasing doctors’ participation in leadership is thought to potentially improve institutional performance, especially when doctors occupy positions of authority within a HI that allow them to participate in managerial decisions (Ham and Dickinson, 2008; Kirkpatrick *et al.*, 2023). On the other hand, only a small number of HIs were led by doctors in the USA (Angood and Birk, 2014) despite evidence showing that leaders with a medical background are beneficial in hospital management (Kirkpatrick *et al.*, 2023). One possible explanation for this situation is that non-medical leaders offer more expertise necessary to effectively lead the HI from the administrative, organizational and financial perspectives because of their background education and training in business or finance (Schwartz and Pogge, 2000). In India, most hospital’s chief executive officers (CEOs) are doctors, but non-medically related management skills such as leadership, team-building, interpersonal skills and communication have not been given due attention (Gayathri and Warrier, 2022). As a result, some postulated that certain leaders of the medical profession were preoccupied with protecting their positions and inept at taking organisational decisions, potentially creating an environment prone to malpractice and corruption (Kumar, 2015). In Malaysia, the Ministry of Health (MOH) is the biggest health-care provider in the country. Under the ministry, a health-care worker, particularly a doctor, is generally the leader of HIs such as hospitals or district health offices. Previously, some of the leaders were appointed as the head of a health-care institution based on their seniority in service and management skills (Mastura, 2008). In recent years, the majority of health-care leaders in the hospital or district health offices would be Public Health Physicians or holds a relevant postgraduate qualification in Master's in Health Management, Master's in Business Administration or Master's in Law (Mohd, 2021; Razak, 2021).

**Figure 1.**
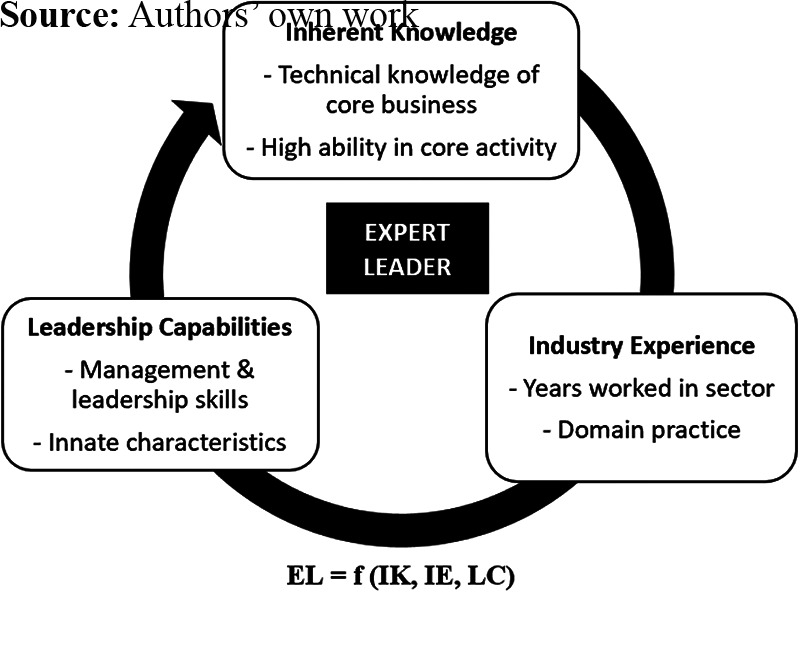
Theory of expert leadership (Goodall, 2016)

The “Theory of Expert Leadership” (TEL) suggests that organizations perform more effectively when led by individuals with a deep understanding of the core business of their organizations (Goodall, 2012). According to TEL (Figure 1), expert leadership (EL) is a function of three factors:

- inherent knowledge (IK), which is obtained through technical knowledge of the core-business activity, attained through education and practice, combined with high ability in the core-business activity;
- industry experience (IE), which equates to time and experience in the core-business industry; and
- leadership capabilities (LC), which includes the experience of management and leadership acquired through education and training (Goodall, 2012).

Thus, leaders’ IK, as well as their industry experience and leadership capabilities, is hypothesized to be positively connected with organizational performance. In other words, TEL supports the evidence that it is necessary to have a health-care professional background to lead a health-care institution. However, with the increasing demands and challenges in health-care systems, the diversity of functions and responsibilities that fall to medical leadership has expanded, necessitating an individual with a broader range of training and competence than merely a senior officer. One of the debated topics in the field of competent leadership is how much core business knowledge the leaders need to have to perform effectively, especially in specialized fields such as health care.

In general, while medical health-care professionals receive specialized training to hone their technical skills, effective leadership training is frequently overlooked. It is critical to have effective leadership at all levels for an organization to obtain better performance (Hogan and Kaiser, 2005). This review aimed to summarize the state of the current literature and to identify gaps that will provide direction for future research in the area of the association between leaders with medical backgrounds and health-care institutional performance. We exclusively looked for quantitative empirical studies reporting on leadership performance that included both medical and non-medical leaders to objectively answer the research question and to minimize the confounders associated with comparisons in health care.

## Material and methods

We conducted a scoping review based on the methodology developed by Arksey and O’Malley (Westphaln *et al.*, 2021) and refined by Levac *et al.*(2015) with enhanced guidance from the *Joanna Briggs Institute Manual* (The Joanna Briggs Institute, 2015). Furthermore, this paper adheres to the Preferred Reporting Items for Systematic Reviews and Meta-Analyses (PRISMA) extension for scoping reviews (Tricco *et al.*, 2018).

### Stages of a scoping review

#### Stage 1: formulating the research question.

In view of the varying performance of HIs under the helm of people with different leadership backgrounds, we intended to establish an understanding of how the dynamic leadership background may influence the performance of HIs. Based on these objectives, the research question of this review is as follows: Are HIs better managed by a leader of medical or non-medical background?

#### Stage 2: identifying relevant studies.

The search strategy was developed by the research team. It included various keywords and relevant synonyms. Based on the research questions, the search terms derived included “performance,” “impact,” “medical,” “physician,” “doctor,” “leader,” “healthcare institutions” and “hospital.” In an iterative process, various combinations of keywords were used in keeping with the scoping review methodology. The final search string was as follows: (performance or impact) and (physician or medical or doctor or leader) and (healthcare institution or hospital). The identification of keywords and the selection of search strings using Boolean logic is important, as it influences the materials that will be retrieved. To qualify as a medical leader, by this study’s criterion, a leader must have been trained in medicine (MD). The inclusion and exclusion criteria were listed below. Only journal articles were considered. While research has been conducted before the year 2000, the purpose of this scoping review was to identify the most recent and relevant articles, thus any older publications before 2000 were excluded.

##### Inclusion criteria:

- Articles published in the English language.
- The study setting was a HI (hospital).
- Quantitative study.
- Published between 2000 and 2022.
- Full text available.

##### Exclusion criteria:

- Articles are written in languages other than English.
- Study setting other than HI (hospital).
- Qualitative study, commentaries, essays, reviews and consensus statements.
- Published before 2000 or after 2022.
- Full texts not available.

A total of three databases were searched, namely, PubMed, Ovid Medline and Google Scholar. These databases were selected based on their relevance to health and human services. In accordance with the standard approach to conducting scoping reviews, a quality appraisal was not performed.

#### Stage 3: selecting the literature.

**Figure 2.**
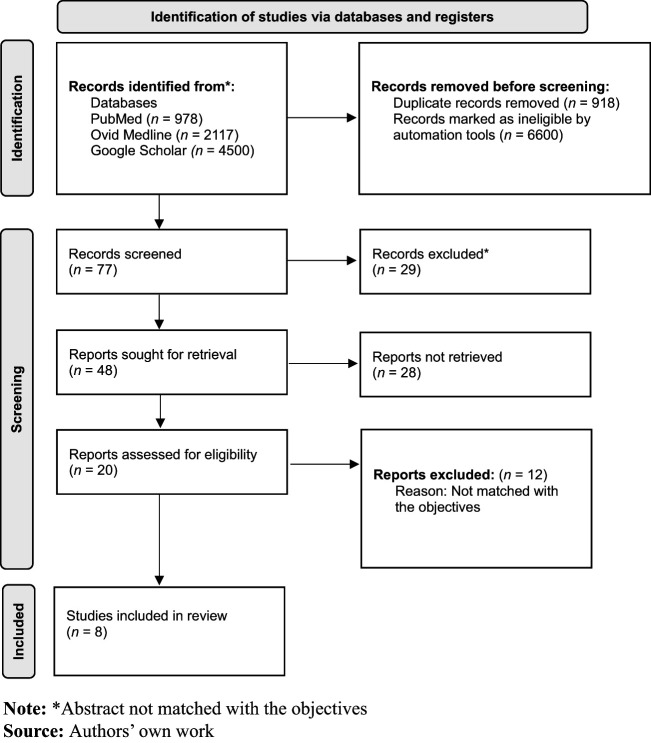
PRISMA flow diagram

In this iterative process, all the retrieved search results and their reference lists were screened based on the predetermined inclusion and exclusion criteria. Two investigators independently screened the titles and abstracts of all retrieved publications for eligibility. Accordingly, the full texts of all publications identified as relevant to the objective of this scoping review were retrieved and reviewed against the same inclusion criteria. If the information provided in either the title and/or the abstract was insufficient for a justified decision, the articles were included in the full-text screening phase. In the event of any disagreements on the inclusion of certain studies between the reviewers, this was resolved by a third reviewer. Subsequently, a PRISMA flow diagram was used to ensure a comprehensive final report for the review completed (Figure 2).

#### Stage 4: charting the data.

In this fourth stage of the scoping review framework, data extracted from the selected articles were entered into Microsoft Excel and analyzed. Charting was done through an iterative process at the early stage of the data extraction. The aim of charting the data was to create a descriptive summary of the results to address the objectives of the scoping review and to answer the research questions. Two investigators extracted the data independently from articles to ensure the accuracy, consistency and comprehensiveness of the data. Any discrepancy in our data extraction was discussed and solved by an agreement. According to the *JBI Reviewer’s Manual* (The Joanna Briggs Institute, 2015), the data charted for each paper should include study objectives, design and outcomes. The authors, objective of the study, study design, outcome and study limitation were summarized in Table 1.

#### Stage 5: collate, summarize and report results.

In the last stage of the framework (Arksey and O’Malley, 2005), the relevant findings were organized into themes. The results were prioritized based on their relevance to the research questions. Pertinent data such as the type of performance indicators and outcomes were outlined (Table 2).

## Results

Out of the 48 studies from full-text screening, 8 studies were included in this review. Another 40 studies were excluded based on the inclusion and exclusion criteria. Most of the studies included were from the USA (Bai and Krishnan, 2015; Goodall, 2011; Mkandawire, 2017; Tasi *et al.*, 2017), followed by two studies from the UK (Veronesi *et al.*, 2013, 2015) and one study each from Germany (Kaiser *et al.*, 2020) and Arab World (Fares *et al.*, 2018). All studies were quantitative and used a comparative cross-sectional study design (leaders with medical background vs non-medical background). Convenience sampling was applied in all studies. Except for two studies, all the remaining studies listed the study limitations (Veronesi *et al.*, 2013, 2015). Table 1 summarizes the studies selected in this review with regard to the association between leaders of medical and non-medical backgrounds with the HI performance.

Table 2 shows a summary of the types of performances and outcomes reported in the selected studies. According to Table 1 and Table 2, all eight studies compared the performance of the HI led by leaders with or without a medical background. However, different types of performances were measured in each study (Table 2) and various outcomes were detected for each performance. Four studies reported a higher performance of HI led by leaders with medical backgrounds (Bai and Krishnan, 2015; Goodall, 2011; Veronesi *et al.*, 2013, 2015) and only one that did not (Fares *et al.*, 2018). In contrast, one study reported no significant difference between the leader’s background and the performance of HIs (Mkandawire, 2017). Finally, two other studies reported a mixed outcome with regard to the association between medical leadership and HI performance (Kaiser *et al.*, 2020; Tasi *et al.*, 2017).

## Discussion

Health-care systems are made up of several different professional groups, departments and specializations that interact in complicated and nonlinear ways. The complexity of such systems is sometimes unparalleled, leading to limitations in different departments with multidirectional objectives and a multidisciplinary workforce. These different groups may either be in support or conflict with one another. Some relevant studies have highlighted that the lack of concordance between hospital employees and management staff can result in conflict, poor decision-making, dissatisfaction and, subsequently, poor patient care standards (Bai and Krishnan, 2015; Goodall, 2011; Tasi *et al.*, 2017; Veronesi *et al.*, 2013). When establishing management procedures, leaders must efficiently use resources while motivating employees to strive toward shared goals to uplift organizational performance. To optimize leadership in these highly complex settings, multifaceted leadership techniques are vital in the health-care setting.

According to TEL, it is necessary to have a health-care professional background to lead a health-care institution. It was observed that the top 100 hospitals in the USA were statistically more likely to be led by medical background instead of non-medical background leaders (“America’s 100 best hospitals, according to Fortune,” 2021). Furthermore, in 2021, it was found that a physician served as CEO at all of the Top 10 Best Hospitals Honour Roll in the USA (Harder, 2021). It has been highly debated for many years whether leaders with or without medical background perform better in the management of HIs. Based on our findings in this review, it was challenging to have a direct comparison in view of the different measures of performance used in each study. However, in general, half of the studies reported a higher performance among HIs led by leaders with a medical background (Bai and Krishnan, 2015; Goodall, 2011; Veronesi *et al.*, 2013, 2015) while one did not (Fares *et al.*, 2018). In addition, two studies reported mixed outcomes (Kaiser *et al.*, 2020; Tasi *et al.*, 2017) and no difference between leaders with or without a medical background was detected in another study (Mkandawire, 2017).

### Management style

The results indicated that in certain instances, being a skilled management leader alone may not be sufficient in ensuring the good performance of HIs. Several factors may explain the phenomenon. In a larger sense, the goals of a HI may be different in the eyes of medical and non-medical leaders. To begin with, the management style of medical leaders is often patient-oriented (Gupta, 2019) with the ultimate aim of improving the quality of care and patient satisfaction. On the other hand, non-medical leaders may lack certain technical expertise and practical understanding of medical management because their training and background typically focus on management principles rather than specific medical knowledge and practices (Goodall, 2011). Leaders with pure management and economic background may be more inclined to focus on overall operating effectiveness as compared to medical leaders who tend to emphasize more on individual patient care more as a result of their medical education.

### Hospital performance: patient satisfaction

With regard to patient satisfaction, it is one of the key performance indicators of quality improvement in HIs. Thus, it is increasingly becoming a critical component to be targeted by health-care leaders in the long-term sustainability and performance of HIs. In the literature, a direct relationship between patient satisfaction and improved health-care quality has been reported (Al-Abri and Al-Balushi, 2014). Furthermore, HIs with higher patient satisfaction scores generally had lower readmission rates (Protomastro, 2016). An increasing level of patient satisfaction may also enhance employee satisfaction. In the UK NHS, promoting leadership from a medical background is seen as a vital component in enhancing institutional performance, mainly because when doctors with clinical experiences hold positions of power within HIs, it allows them to participate in and contribute to important management-related decisions (Ham and Dickinson, 2008). Apart from that, the advantages of appointing doctors to health-care administrative positions include more effective bottom-up leadership and improved communication with top management (Loh, 2015). A previous study reported that competent health-care leaders can positively influence patient satisfaction by strengthening cooperation through employee teamwork, mutual support and communication (Bruning, 2013).

### Hospital performance: financial performance

Despite most of the studies supporting the advantages of medical leadership in the performance of HIs, one study in this review reported a poorer financial performance among HIs led by medical leaders (Kaiser *et al.*, 2020). Many believed that the non-medical leadership model tends to reorient a hospital's goal away from patient care toward profitability (Gupta, 2019). While the poor financial performance of a HI might not be seen as a critical problem for countries with a heavily subsidized health-care system and non-profit-driven health-care services, for many other profit-driven private HIs, the ability to create profit is crucial in increasing operational efficiency and ensuring sustainability. HIs, particularly hospitals, require substantial financial assistance to offer patients with high-quality facilities and services. However, there is a lack of other studies to support the correlation between hospital financial performance and the academic background of the leader. Further investigation that uses a bigger sample size and more robust financial performance indicators is warranted.

### Hospital performance: a Web-based perspective

In this review, the only study that reported that medical leadership was significantly associated with a lower HI performance based on hospital ranking was from the Arab World (Fares *et al.*, 2018). However, the performance measured only one particular hospital ranking that was based on web indicators through visibility, size, rich files and “scholars.” Web indicator or “Web Impact Factor” in this study was based on a link analysis that combined the number of external links (visibility), the number of pages of the website (size) (Almind and Ingwersen, 1997), the number of documents measured from the number of rich files in a Web domain (rich files) and the number of publications being collected by Google Scholar database (“scholar”). The four indicators were obtained from the quantitative results provided by the main search engines. In other words, the hospital activity was merely measured based on the web presence. Therefore, it remains inconclusive if non-medical leaders are more effective leaders than medical leaders in an actual setting.

### Leadership effectiveness: task-relevant qualifications

On a different note, one of the studies in this review reported that there were no significant differences between non-medical and medical leaders in terms of hospital net income, patient experience ratings and mortality rates (Mkandawire, 2017). The study postulated that competent leaders often adapt their leadership styles based on the maturity of the individuals or groups that they are attempting to lead or influence, in line with the ‘Situational Leadership Theory (Graeff, 1983). Thus, a good leader can rise to the leadership role, irrespective of their background, proving that “effective leadership is task-relevant” (Graeff, 1983). The findings of this study put forth the argument about who is more qualified to run the HI on a social level. In short, it is very important to determine the right person with the necessary credentials, passion and capacity to take on the responsibility of the leadership role.

Although many health-care professionals acknowledge the benefits of medical leaders and the qualities they possess, they also believe that most health-care workers lack the necessary knowledge of leadership skills (Chen, 2018; Ghiasipour *et al.*, 2017). While the 70:20:10 model of leadership development highlights that most learning results from experiences and relationships and only 10% from formal training (Blackman *et al.*, 2016), there is a beneficial need for formal training to strengthen significant leadership competencies among medical leaders that can complement and enhance experiential learning and developmental relationships. However, medical management specialization has advanced tremendously globally in recent years. Most of the courses share the core concepts of merging medical knowledge and skill with management and health-care training. This is especially true for financial management as HIs should focus on efficient cost management to ensure the sustainability of health-care financing. Yet, this process is complicated since cost-cutting efforts require the collaboration of all members in the HIs led by the centralized management team. As a result, it is imperative for HIs to be helmed by leaders who can strike a balance between effective financial management and patient care delivery without compromising the health and quality of patients. In line with this, the quality of patient care should be a fundamental performance dimension to be assessed in future studies and multidimensional constructs of quality should be considered, as it cannot be fully by just one or two indicators as illustrated by the Donabedian framework (NHS, 2018).

### Limitations

The limitations of this review are mainly because of the nature of the scoping technique, such as the lack of quality appraisal for included studies and the potential for interpretation bias. We also had to strike a balance between comprehensiveness and feasibility. Besides, we might have missed out on some relevant research, such as those conducted in another language, in a different database or in a non-quantitative study. There were no studies that could reflect the experience of low- and middle-income countries (LMIC). Finally, even though a scoping review was appropriate for our main objective of determining whether HI is better managed by a leader with a medical or non-medical background, we acknowledge that it is difficult to make a fair comparison because of the varied types of performance measures between studies.

## Conclusion

Like medicine, the field of management and leadership requires ongoing refinement and adaptation with the necessary skills, dedication, education and experience. In general, based on the review findings, medical professionals lead to better performance of HI, likely because they are in a position to shape HI policies that align with the core philosophy of “patient-first.” However, it is imperative to equip medical leaders with essential management abilities to optimize their leadership styles in these highly complex health-care settings. Regardless of their career stage or pathway, it will be beneficial to provide training to strengthen leadership competencies among medical leaders. Finally, further evidence in the form of peer-reviewed studies is warranted, especially from LMIC, to establish a clearer link between the performance of HI led by medical and non-medical leaders.

## Figures and Tables

**Table 1.** A summary of studies included in the review

No.	Author/source/year	Country	Objective	Study design	Outcome	Study limitation
1	Amanda H. Goodall *Social Science and Medicine*, 2011 Goodall (2011)	US	To compare the ranked quality between hospitals led by CEOs who are physicians and those who are non-physician managers	Method: Cross-sectional Location: Top 100 US hospitals in 2009	The majority of CEOs, i.e. 16 out of 21, were physicians A strong positive association between the ranked quality of a hospital and whether the CEO was a physician or not. However, longitudinal inquiries are recommended to establish that physician-leaders improve the performance of hospitals compared to professional managers. Other important variables, such as a CEO’s tenure and the level and number of years of clinical experience that each CEO had obtained are important factors	Cross-sectional analyses cannot be used to infer causality because a temporal sequence cannot be established
2	Gianluca Veronesi *et al.* *Social Science and Medicine Journal*, 2013 (Veronesi *et al.*, 2013)	UK	To determine the impact of clinician appointment to the boards of directors of the NHS hospital trusts	Method: Cross-sectional Location: NHS hospitals in England	The analysis reveals a significant and positive association between a higher percentage of clinicians on boards and the quality ratings received by the service providers. This positive influence is also manifested in lower morbidity rates. Analysis results excluded the possibility of reverse causality (doctors joining boards of already successful organizations)	Not stated
3	Michael C. Tasi *et al.* *Health Care Management Review*, 2017 Tasi *et al.* (2017)	US	To examine whether hospital systems led by physicians were associated with better US news and world report (USNWR) quality ratings, financial performance and operating efficiency as Compared with those led by non-physician managers	Method: Cross-sectional Location: US hospitals	Large hospital systems led by physicians received higher USNWR ratings and bed usage rates than did hospitals led by non-physicians. However, there was no difference in financial performance The results imply that physician leaders may possess skills, qualities, or management approaches that positively affect hospital quality and the value of care delivered	Other confounders may affect the correlation between leadership and hospital quality but certain characteristics were hard to obtain for the analysis. Leaders were only categorized based on their medical degree and other potentially relevant characteristics such as prior health-care administration education and experience, other advanced degrees, or tenure in the health-care industry were not assessed
4	Collins Yazenga and Mkandawire Dissertation PhD Walden University, 2017 Mkandawire (2017)	US	To examine Whether physician or non-physician CEOs perform better in US hospitals based on hospital net income, patient experience ratings and mortality rates	Method: Cross-sectional study Location: 60 US hospitals	No significant differences between hospitals’ net income, patient experience ratings, or mortality rates in hospitals led by non-physician and physician CEOs. Thus, physician and non-physician CEOs may produce similar outcomes and hospital boards can view CEO applicants equally	This process of data compilation could affect the data integrity due to the potential inaccuracies in the reporting of hospital-related mistakes. Convenience sampling methodology was used so the variables in the study were predefined by environmental course
5	Florian Kaiser *et al.* *Social Science and Medicine*, 2020 Kaiser *et al.* (2020)	Germany	To examine the link between the educational background of a hospital's CEO and hospital performance in terms of medical quality and financial success	Method: Cross-sectional Location: 370 German hospitals	Physician-led hospitals have significantly lower in-hospital mortality rates for pneumonia and higher patient satisfaction In contrast, institutions led by managers with economics or business degrees showed better financial performance and superior outcomes for hip and knee surgeries The findings support prior results regarding financial outcomes and mortality	The broad spectrum of measures for clinical quality in the study meant that a straightforward interpretation that physician CEOs lead to superior medical quality could not be conclusively established A considerable number of hospitals were excluded due to missing data
6	Youssef Fares *et al.* *Surgical Neurology International*, 2018 Fares *et al.* (2018)	Arab world	To explore whether hospitals led by physician leaders perform better than hospitals led by non-physician managers	Method: Cross-sectional Location: Hospitals in Arab World	Physician leadership was significantly associated with lower hospital ranking (bottom 50 hospitals) in the Arab World	Only one hospital quality indicator was used for ranking. For better evaluation, the ranking system must also focus on patient satisfaction and perception of quality to evaluate the impact of medical leadership
7	Ge bai and Ranjani Krishnan *American Journal of Medical*, 2015 Bai and Krishnan (2015)	US	To examine whether hospitals without physician participation on their boards of directors deliver lower Quality of care	Method: Cross-sectional Location: California non-profit hospitals	The lack of physician representation on hospital boards is associated with lower quality of care In other words, physicians as directors add important value to hospital quality of care	Only confined to one state, California The data obtained from the hospital quality alliance (HQA) program included only major medical conditions that might not accurately reflect the overall hospital quality of care Self-reported quality of care data by hospitals can have data manipulation
8	Gianluca Veronesi *et al.* *Public Administration*, 2015 Veronesi *et al.* (2015)	UK	Does increased participation of clinical professionals on hospital boards impact positively performance outcomes (patient experience)?	Method: Cross-sectional Location: Acute hospital sector in the NHS	Clinical participation on hospital governing boards significantly improved the patient experience of the care provided	Not stated

**Source:** Authors’ own work

**Table 2.** A summary of the type of performances and outcomes of the studies in the review

Author	Type of performances	Outcome of medical leadership
Amanda H. Goodall (Goodall, 2011)	Hospital quality ranking: i. Patient care ii. Delivery of care iii. Mortality rates	Higher performance
Gianluca Veronesi *et al.* (Veronesi *et al.*, 2013)	Hospital quality ranking: i. Health and well-being ii. Clinical effectiveness iii. Safety and patient focus iv. Ease and equity of access	Higher performance
Ge Bai and Ranjani Krishnan (Bai and Krishnan, 2015)	Quality of care	Higher performance
Gianluca Veronesi *et al.* (Veronesi *et al.*, 2015)	Patient experience	Higher performance
Michael C. Tasi *et al* (Tasi *et al.*, 2017)	Hospital quality ranking: i. Patient care ii. Delivery of care iii. Mortality rates	Higher performance
	Hospital volume	No difference
	Financial performance	No difference
Collins Yazenga and Mkandawire (Mkandawire, 2017)	Hospital net income Patient experience ratings Mortality rates	No difference
Florian Kaiser *et. al.* (Kaiser *et al.*, 2020)	Mortality rates Patient satisfaction	Higher performance
	Financial performance	Lower performance
Youssef fares *et. al*. (Fares *et al.*, 2018)	Hospital ranking – Web indicator based on visibility, size, rich files and scholar	Lower performance

Notes:

HI = health-care institution; NHS = National Health Service; MOH = Ministry of Health; UK = United Kingdom; US = United States; TEL = theory of expert leadership; EL = expert leadership; IK = inherent knowledge; IE = industry experience; LC = leadership capabilities; PRISMA = Preferred Reporting Items for Systematic Reviews and Meta-Analyses; MREC = Medical Research and Ethics Committee; MD = medicine; CEO = chief executive officer; HQA = Hospital Quality Alliance; NHS = National Health Service; LMIC = low- and middle-income countries

**Source:** Authors’ own work
